# From homeostasis to neurodegeneration: Müller glia in retinal disease

**DOI:** 10.3389/fcell.2026.1980008

**Published:** 2026-10-29

**Authors:** Matangi Kumar, Shruthi Karnam, John G. Flanagan, Karsten Gronert

**Affiliations:** 1 Vision Science Graduate Group, University of California Berkeley, Berkeley, CA, United States; 2 Herbert Wertheim School of Optometry and Vision Science, University of California Berkeley, Berkeley, CA, United States; 3 Infectious Disease and Immunity Graduate Group, University of California, Berkeley, Berkeley, CA, United States

**Keywords:** glaucoma, gliosis, Müller glia, neurodegeneration, neuroinflammation, retina

## Abstract

Müller glia are the principal macroglia cells of the retina and are essential for maintaining structural, metabolic, synaptic, and vascular homeostasis. They regulate neurotransmitter recycling, ion and water balance, antioxidant defense, and neurovascular coupling, positioning them as central coordinators of retinal function in health and disease. In response to injury or stress, Müller glia transition from homeostatic support to reactive states that may initially preserve tissue integrity but, when sustained, contribute to chronic neuroinflammation, barrier dysfunction, and neuronal degeneration. In this review, we examine the core physiological functions of Müller glia and highlight emerging evidence that their responses are heterogeneous, reflecting spatial, molecular, and disease-specific differences across retinal layers and pathological contexts. We discuss major drivers of Müller glia activation, including mechanical stress, metabolic and oxidative imbalance, and inflammatory signaling, with emphasis on convergent pathways such as gp130–JAK–STAT3, NFκB, and mechanosensitive calcium signaling. We then consider how disruption of Müller glia homeostatic functions contributes to retinal disease, focusing on glaucoma, diabetic retinopathy, and age-related macular degeneration. Across these disorders, Müller glia emerge not as passive support cells, but as active regulators of neuronal and vascular microenvironments whose state of transition may shape disease progression. Finally, we discuss therapeutic strategies aimed at modulating Müller glia states, including lipid metabolism and signaling, restoration of ion and metabolic homeostasis, gene-based targeting, and regenerative reprogramming, and consider how single-cell, spatial, and multi-omic approaches define distinct layers of Müller glia state. Defining when and how Müller glia shift from adaptive to maladaptive states will be critical for developing therapies that restore retinal homeostasis and limit neurodegeneration.

## 1 Introduction

The retina is a highly specialized central nervous system (CNS) tissue that converts light into neural signals (Nguyen et al., 2021). While numerous neuronal subtypes contribute to visual processing in the human retina, increasing evidence has highlighted the essential role of retinal glia in maintaining tissue homeostasis, coordinating responses to injury, and shaping neuroinflammatory signaling (García-Bermúdez et al., 2021).

The vertebrate retina contains three principal glia subtypes: astrocytes, microglia, and Müller glia. Among these, Müller glia represent the predominant macroglia population (astrocytes and Müller glia), comprising approximately ninety percent of retinal glia and uniquely deriving from retinal progenitor cells (Tworig and Feller, 2021; MacDonald et al., 2017; Vecino et al., 2016; Reichenbach and Bringmann, 2013). They provide structural support, regulate ion and water balance, recycle neurotransmitters, and contribute to blood–retinal barrier function (Li X. et al., 2019; Peña and Vazquez, 2022; Kolb, 1995). Through extensive interactions with neurons and vasculature, Müller glia coordinate structural stability, metabolic support, and vascular integrity across retinal layers (Li X. et al., 2019; Tout et al., 1993).

Müller glia are therefore uniquely positioned to regulate the retinal microenvironment. In response to stress, they undergo transitions from homeostatic support to reactive states that can initially preserve tissue integrity but, when sustained, contribute to chronic inflammation and neurodegeneration. Defining the mechanisms that govern these state transitions is critical for understanding disease progression and identifying therapeutic opportunities.

In this review, we examine how Müller glia homeostatic functions are organized across the healthy retina and how these functions become altered in disease. We then discuss key drivers of Müller glia activation, the consequences of sustained reactivity across major retinal disorders, and emerging strategies to therapeutically modulate Müller glia states, including the lipid and metabolic cues that may set their threshold for reactivity.

## 2 Cellular architecture and homeostatic functions of Müller glia

### 2.1 Development of Müller glia

In contrast to retinal astrocytes, which enter the retina through the optic nerve during development, and microglia, which originate outside the neuroepithelium and later colonize the tissue, Müller glia arise from multipotent retinal progenitor cells and share lineage with retinal neurons (Tworig and Feller, 2021; Paisley and Kay, 2021; Li F. et al., 2019). Their differentiation occurs late in retinogenesis, alongside rod photoreceptors and bipolar cells, after the major retinal layers and synaptic architecture have largely been established (Tworig and Feller, 2021; Jadhav et al., 2009) (Figure 1A).

**FIGURE 1:**
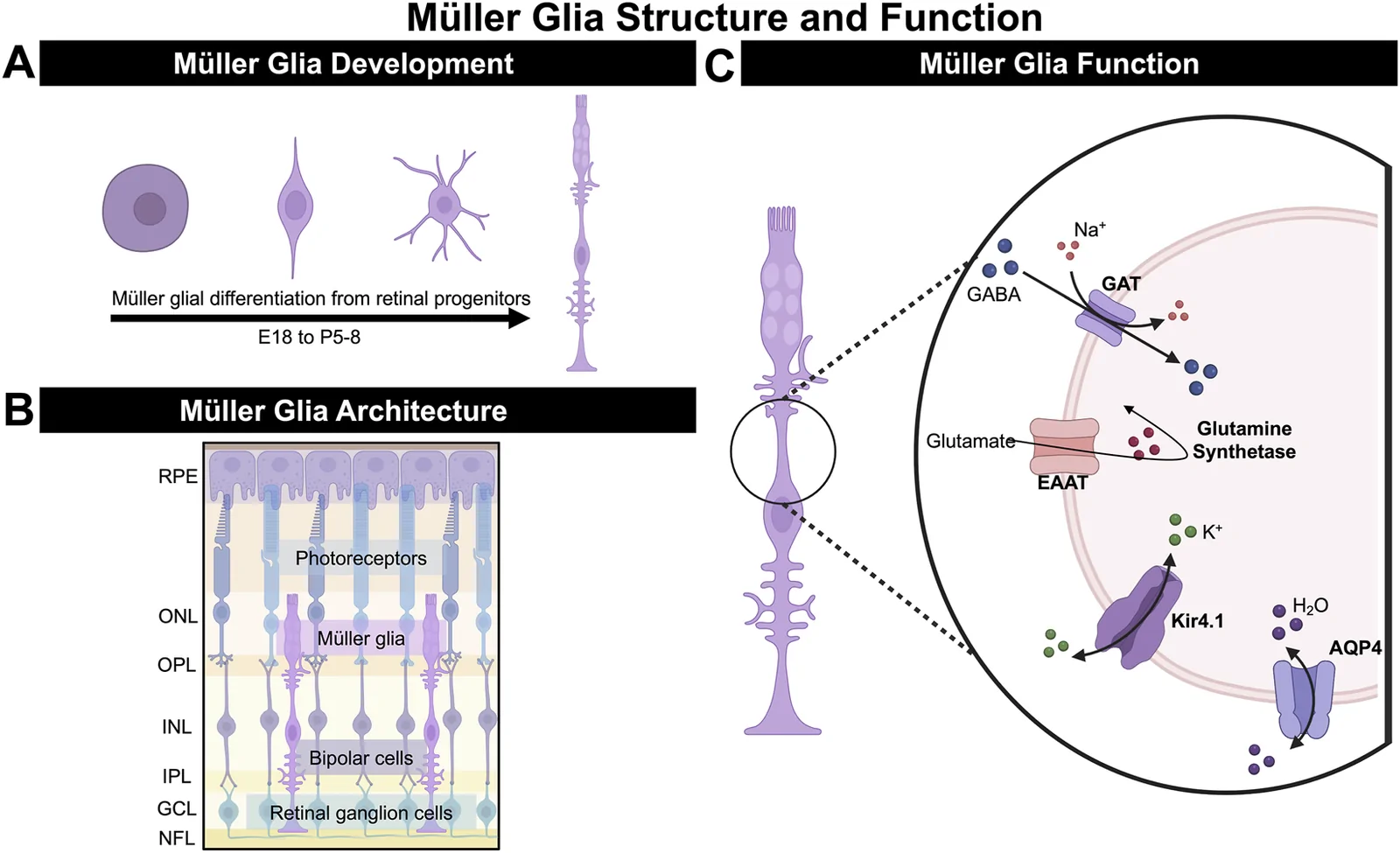
Development, retinal architecture, and core homeostatic functions of Müller glia. **(A)** Schematic of Müller glia development in the mouse retina. Müller glia arise from late-stage retinal progenitor cells and differentiate during late embryonic and early postnatal development, beginning around embryonic day 18 (E18) and continuing through approximately postnatal day 5–8 (P5–P8). **(B)** Schematic of Müller glia architecture within the layered retina. Müller glia extend radially across the neural retina, spanning from the outer to inner retinal compartments and interacting closely with retinal neurons throughout multiple laminae. Abbreviations: RPE, retinal pigment epithelium; PL, photoreceptor layer; ONL, outer nuclear layer; OPL, outer plexiform layer; INL, inner nuclear layer; IPL, inner plexiform layer; GCL, ganglion cell layer; NFL, nerve fiber layer. **(C)** Simplified schematic of representative Müller glia functions. These include neurotransmitter handling through GABA uptake and glutamate clearance, glutamine synthesis, potassium buffering mediated in part by Kir4.1, and water homeostasis associated with AQP4 (García-Bermúdez et al., 2021; Tworig and Feller, 2021; Grimes et al., 2025; Wang et al., 2017).

This late developmental timing is functionally important. Unlike radial glia populations that guide neuronal migration during early neurogenesis, Müller glia mature within an already organized retinal circuit (Tworig and Feller, 2021; Jadhav et al., 2009). They are therefore positioned not to build the initial retinal scaffold, but to stabilize established neuronal architecture at a stage when synaptic activity and metabolic demand are increasing. As they mature, Müller glia assume key roles in maintaining extracellular homeostasis and supporting reliable neurotransmission.

### 2.2 Müller glia morphology and architecture

Müller glia are radially oriented cells whose processes extend through the synaptic and nuclear layers. Their microvilli in the outer retina contact photoreceptor inner segments and contribute to the outer limiting membrane, while their cell bodies reside in the inner nuclear layer and fine processes course through the plexiform layers to interact with neuronal synapses (MacDonald et al., 2017; Kolb, 1995; Grimes et al., 2025). At the vitreal surface, Müller glia endfeet ensheathe neurons and retinal vessels, providing an anatomical basis for neurovascular signaling and contributions to blood–retinal barrier organization (Tout et al., 1993; Grimes et al., 2025).

Müller glia are also not morphologically uniform. Layer-specific analyses reveal sublaminar specialization, particularly within the inner plexiform layer, and regional differences are evident between primate foveal and peripheral retina (MacDonald et al., 2017; Grimes et al., 2025; Jubran et al., 2025). Together, these findings indicate that Müller glia architecture is functionally specialized and shaped by local synaptic, metabolic, and vascular demands. This structural organization is important in disease because even subtle remodeling of Müller glia processes may alter neuronal, extracellular, and vascular interactions (Figure 1B).

### 2.3 Neurotransmitter homeostasis

Müller glia regulate extracellular neurotransmitter levels to maintain retinal synaptic signaling and circuit stability. They clear the primary excitatory neurotransmitter, glutamate, via high-affinity transporters (EAAT1/GLAST) to terminate postsynaptic signaling and prevent excitotoxic stress (García-Bermúdez et al., 2021; Bringmann et al., 2013). Internalized glutamate is then converted to glutamine by glutamine synthetase (GS) and returned to neurons to sustain the glutamate–glutamine cycle (Bringmann et al., 2013; Oyetayo et al., 2025).

Disrupting this pathway can compromise retinal function. Reduced GS or glutamate transporter activity limits substrate availability for bipolar and ganglion cells and can cause extracellular glutamate accumulation (García-Bermúdez et al., 2021; Oyetayo et al., 2025). Elevated glutamate can also promote oxidative stress by inhibiting cystine uptake and reducing intracellular glutathione, increasing vulnerability to reactive oxygen and nitrogen species during ischemia (Bringmann et al., 2013; Oyetayo et al., 2025).

Müller glia also maintain inhibitory tone through GABA uptake, preventing neurotransmitter spillover between synapses (García-Bermúdez et al., 2021; Bringmann et al., 2013). Notably, GABA signaling in Müller glia is linked to neurovascular pathways, including the modulation of vascular endothelial growth factor (VEGF) expression, suggesting that neurotransmitter handling helps coordinate neuronal activity with vascular stability (Medina-Arellano et al., 2026). By governing the spatial and temporal dynamics of both excitatory and inhibitory signaling, Müller glia act as central stabilizers of retinal circuits, a role that extends to the regulation of extracellular ion and water balance (Figure 1C).

### 2.4 Ion and water homeostasis

Neuronal activity in the retina depends on tightly regulated extracellular ion and water balance. Müller glia support this activity through spatial potassium buffering and water movement, enabled by inwardly rectifying potassium channels such as Kir4.1. (Kir4.1) and by aquaporin-4 (AQP4) respectively, which are enriched in specialized membrane domains (Peña and Vazquez, 2022; Han et al., 2024). Experimental disruption of Kir4.1 alters potassium handling and changes electroretinogram responses, including reductions in b-wave components and altered slow potentials that reflect glia contributions to retinal signaling (Han et al., 2024; Reichenbach et al., 2007). In parallel, dysregulation of AQP4 and Kir4.1 has been linked to retinal edema and impaired retinal function in stress models, supporting the concept that osmotic balance and ionic buffering are tightly coupled to tissue-level performance (Han et al., 2024; Reichenbach and Bringmann, 2020).

Taken together, these findings indicate that Müller glia ion and water regulation is not simply passive support. By stabilizing extracellular potassium and osmotic balance, Müller glia preserve the conditions required for reliable neuronal signaling, resistance to edema, and maintenance of retinal tissue integrity (Figure 1C).

## 3 Müller glia and the inner blood-retinal-barrier

### 3.1 Structural organization of the retinal neurovascular unit

Beyond regulating synaptic and ionic homeostasis, Müller glia also help coordinate vascular stability and metabolic exchange within the retinal neurovascular unit. The retina is among the most metabolically active tissues in the body and therefore depends on tightly regulated blood flow and barrier function to meet local oxygen and glucose demand while preserving optical transparency (O’Leary and Campbell, 2023; Bora et al., 2023; Runkle and Antonetti, 2011). This is achieved through a dual circulation system and the blood–retinal barrier (BRB). The outer retina is supplied primarily by the choroidal circulation and separated from it by the outer BRB, which is formed by the retinal pigment epithelium. In contrast, the inner retina is supplied by the retinal circulation and protected by the inner BRB, which is established by tight junctions between retinal endothelial cells and supported by the surrounding neurovascular unit, including pericytes, glia, and immune cells (Runkle and Antonetti, 2011; Claude, 1963; Yu et al., 2010; Figure 2).

**FIGURE 2:**
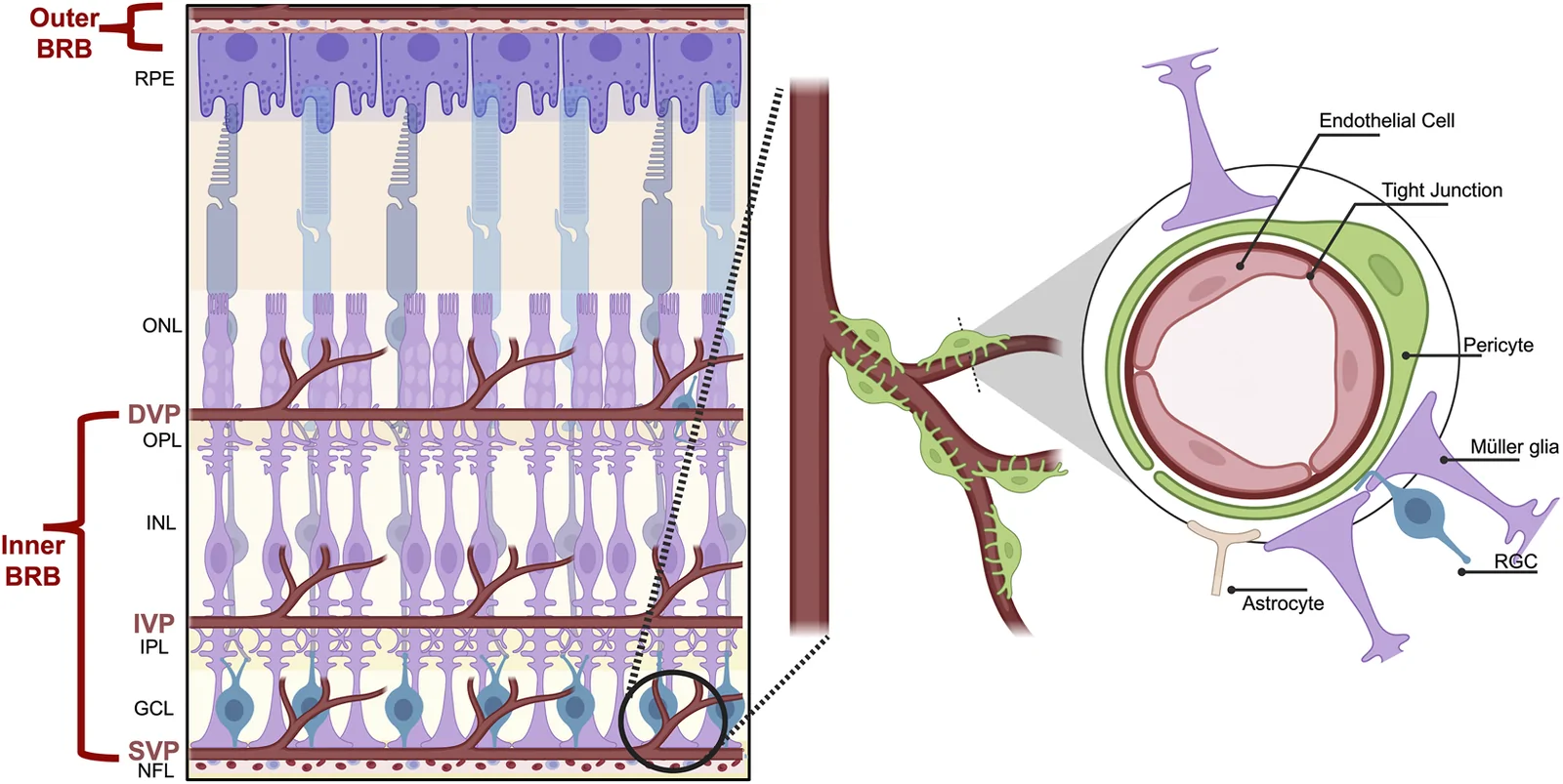
Müller glia and the retinal neurovascular unit and blood-retinal barrier. Schematic illustrating the position of Müller glia within the layered retina and their association with the outer and inner blood-retinal barriers. The outer blood-retinal barrier is formed by the retinal pigment epithelium (RPE), whereas the inner blood-retinal barrier is established by retinal vascular endothelial cells linked by tight junctions and supported by pericytes and glia cells. Müller glia endfeet contact vessels within the superficial (SVP), intermediate (IVP), and deep vascular plexuses (DVP). The magnified inset highlights the inner retinal neurovascular unit, in which Müller glia endfeet closely appose endothelial cells and pericytes, together with astrocytes and adjacent retinal ganglion cells (RGCs). Abbreviations: BRB, blood-retinal barrier; RPE, retinal pigment epithelium; ONL, outer nuclear layer; OPL, outer plexiform layer; INL, inner nuclear layer; IPL, inner plexiform layer; GCL, ganglion cell layer; NFL, nerve fiber layer; RGC, retinal ganglion cell (Li X. et al., 2019; Tout et al., 1993; Grimes et al., 2025)

Within this system, glia–vascular interactions are layer-specific. Astrocytes are associated primarily with vessels in the superficial vascular plexus, whereas Müller glia provide the principal glia contribution to vascular interactions in deeper retinal layers. Müller glia processes and endfeet closely appose capillaries and synaptic layers, positioning them to coordinate neuronal activity with vascular permeability and blood flow (Bora et al., 2023; Claude, 1963; Coorey et al., 2012). Recent ultrastructural studies further suggest that Müller glia vascular sheathing is compartmentalized across vascular plexuses, with nearly complete ensheathment in the superficial plexus and more fenestrated organization in deeper layers, supporting a model in which Müller glia shape where and how neurovascular signaling occurs across the inner retina (Grimes et al., 2025; Coorey et al., 2012; Figure 2).

### 3.2 Regulation of the inner blood–retinal barrier

Under homeostatic conditions, Müller glia contribute to vascular stability by supporting the neurovascular unit through structural interactions and paracrine signaling. Although barrier function is established primarily by endothelial tight junctions, Müller glia help regulate endothelial stability and permeability through gap junction–associated communication and through secretion of factors that shape angiogenic balance (Medina-Arellano et al., 2024). A major pathway in this process is VEGF signaling. Müller glia, together with other retinal cells, produce VEGF under developmental and stress conditions, while also expressing anti-angiogenic factors such as pigment epithelium-derived factor and thrombospondins (Peña and Vazquez, 2022). Because VEGF signaling is highly context-dependent, shifts in the balance between pro- and anti-angiogenic cues can promote either vascular maintenance or vascular instability. This helps explain why Müller glia regulation of VEGF pathways is important in development and remains a major determinant of barrier function in disease.

### 3.3 Neurovascular dysfunction and barrier breakdown

When the balance of neurovascular signaling is disrupted by hypoxia, ischemia, oxidative stress, or chronic inflammation, the retinal neurovascular unit shifts toward barrier compromise, vascular leakage, and, in severe cases, pathological neovascularization. Müller glia are central to this transition because they respond rapidly to metabolic stress and alter the local profile of angiogenic and inflammatory mediators (Vecino et al., 2016; Peña and Vazquez, 2022). Under hypoxic or ischemic conditions, Müller glia upregulate VEGF and other permeability-associated signals, and conditional genetic studies show that Müller glia–derived VEGF is a major driver of vascular leakage and inflammatory lesions in retinal vascular disease models (Yu et al., 2010; Medina-Arellano et al., 2024; Kumari et al., 2025; Le, 2017). Additional mediators, including fibroblast growth factor 2 (FGF2) and inflammatory cytokines such as TNFα, further amplify endothelial dysfunction and remodeling (Carpi-Santos et al., 2022). Reactive and proliferative glia responses can therefore destabilize the inner BRB not only by responding to endothelial injury, but by actively reshaping the local balance of barrier-stabilizing and permeability-promoting signals. These mechanisms are especially relevant in diseases such as diabetic retinopathy and ischemic retinal injury, in which chronic Müller glia dysfunction is thought to contribute to vascular pathology (Peña and Vazquez, 2022; Balzamino et al., 2024; Zhou et al., 2025).

### 3.4 Müller glia–retinal ganglion cell metabolic coupling

Müller glia support retinal function not only by maintaining vascular stability but also by sustaining neuronal survival under metabolic stress. By regulating extracellular potassium and water balance, recycling neurotransmitters, and providing metabolic substrates, Müller glia stabilize the extracellular environment required for sustained retinal ganglion cell (RGC) signaling (Zhou et al., 2025). This glia–neuron metabolic coupling becomes especially important during energetic challenge, when RGCs experience reduced substrate availability and increased oxidative stress. Under ischemic conditions, neurons release activity-dependent signals that engage Müller glia and shift glia physiology toward stress-responsive states, altering glia–neuron communication and potentially reshaping circuit output (Vecino et al., 2016; Kumari et al., 2025; Zhou et al., 2025).

These observations support the view that Müller glia act as central metabolic intermediaries linking retinal activity with vascular supply. Although the mechanisms governing Müller glia–RGC coupling, and how they are altered during stress, remain incompletely defined, clarifying these pathways will be important for understanding why RGCs are particularly vulnerable in neurodegenerative retinal disease. Because these neurovascular and metabolic support functions are highly sensitive to mechanical, inflammatory, and oxidative stress, they also provide a useful framework for understanding the inputs that drive Müller glia activation (Peña and Vazquez, 2022; Zhou et al., 2025; Rombaut et al., 2023).

## 4 Mechanisms of Müller glia activation

Following retinal stress or injury, Müller glia are among the earliest responders and rapidly shift from homeostatic support toward reactive states that initially aim to preserve tissue integrity (Graca et al., 2018). This response, broadly termed gliosis, can be adaptive in early stages by stabilizing the extracellular environment, limiting damage spread, and supporting neuronal survival. However, when reactive programs are sustained or excessive, gliosis becomes maladaptive and contributes to chronic neuroinflammation, synaptic dysfunction, and progressive neurodegeneration (Graca et al., 2018; Seitz et al., 2013).

Müller glia activation is triggered by diverse inputs, including mechanical strain, metabolic imbalance, inflammatory cytokines, and disrupted neuronal activity. A commonly used hallmark of Müller glia reactivity is the induction of intermediate filament proteins, particularly glial fibrillary acidic protein (GFAP), vimentin, and nestin (Graca et al., 2018; Bringmann and Wiedemann, 2011). In more severe or persistent conditions, these changes may also be accompanied by architectural remodeling, hypertrophy of glia processes, and, in some contexts, glia scar formation that alters neuronal and vascular interactions (Table 1).

**TABLE 1:** Representative inputs, sensing pathways, and response programs involved in Müller glia activation.

Input category	Key signals and sensors	Müller glia response
Mechanical	Elevated IOP, tissue stretch, cell swelling, shear stress; sensed through TRP channels, piezo channels, and integrin–ECM signaling	Calcium influx, MAPK/ERK activation, reactive gliosis, cytoskeletal and extracellular matrix remodeling, and release of inflammatory mediators and growth factors
Oxidative and metabolic	Hypoxia, ROS, NADPH oxidase activity, mitochondrial dysfunction, excitotoxic or metabolic stress, neurotoxins	Reactive gliosis, Nrf2 activation and glutathione-associated antioxidant responses, metabolic reprogramming with increased glycolytic dependence, impaired glutamate homeostasis, cytokine release, and in severe states, dedifferentiation or dysfunction
Inflammatory and immune	Cytokines, chemokines, NF-κB and JAK/STAT signaling, VEGF, inflammatory lipids, complement, and microglia activation	Reactive gliosis, chemokine and cytokine release, immune cell recruitment and glia–immune crosstalk, ROS/NO production, structural remodeling, vascular/barrier dysfunction, and proliferation

Müller glia respond to diverse retinal stressors that can be broadly grouped into mechanical, oxidative/metabolic, and inflammatory/immune categories. This table highlights representative mediators and sensors associated with each class of input, together with common downstream Müller glia responses. These include reactive gliosis, calcium-dependent signaling, antioxidant and metabolic reprogramming, cytokine and chemokine release, structural remodeling, and altered vascular or barrier support. While shown separately for clarity, these pathways are highly interconnected in retinal disease and often act in combination to shape the magnitude and outcome of Müller glia reactivity (Lundkvist et al., 2004; Jo AO. et al., 2022; Miguel-López et al., 2025; Kirsch et al., 2010; Coorey et al., 2015; Ogundele et al., 2013; Hagemann et al., 2023).

### 4.1 Mechanical stress and mechano-transduction

Anchored at the inner and outer limiting membranes, Müller glia are highly sensitive to biomechanical forces such as intraocular pressure (IOP) strain, osmotic swelling, and extracellular matrix changes (Prieto-López et al., 2024; Lundkvist et al., 2004; Lindqvist et al., 2010). They translate mechanical deformation into state transitions via mechanosensitive ion channels (notably TRPV4 and Piezo channels) and integrin-mediated adhesion signaling (Jo AO. et al., 2022; Jo et al., 2015). A primary consequence of this mechanical activation is increased intracellular calcium, which alters volume regulation and engages kinase programs that drive gliosis, structural remodeling, and the release of inflammatory and vasoactive mediators (Lindqvist et al., 2010; MacDonald et al., 2015; Cullimore et al., 2023). In intact tissue, distinguishing direct Müller glia mechanosensing from secondary signals originating from stressed neurons and vascular cells remains challenging. Because mechanical strain often coincides with energetic challenge and oxidative stress in retinal disease, these pathways are closely linked to metabolic stress responses.

### 4.2 Metabolic and oxidative stress

Müller glia support retinal homeostasis through metabolic buffering and antioxidant defense, but these systems are highly sensitive to hypoxia, reactive oxygen species (ROS) accumulation, and mitochondrial dysfunction. The retina operates under high oxidative demand due to intense oxygen consumption and continuous light-driven activity, with oxygen tension fluctuating across light and dark conditions (Bringmann and Wiedemann, 2011; Miguel-López et al., 2025). In response, Müller glia provide antioxidant support, including glutathione production and recycling, to protect neurons and maintain neurotransmitter handling (Toft-Kehler et al., 2016). When oxidative stress is induced experimentally, for example, through hydrogen peroxide exposure, nutrient deprivation, or hypoxic challenge, Müller glia exhibit reduced mitochondrial respiration and impaired glutamate transport capacity, alongside increased expression of reactive markers and activation of hypoxia-responsive pathways (Miguel-López et al., 2025; Toft-Kehler et al., 2016). Under these conditions, Müller glia may also exhibit osmotic swelling and altered membrane homeostasis, linking metabolic stress to structural and physiological changes that contribute to reactive state transitions. These metabolic and redox disturbances also intersect with lipid handling, which we return to in Section 6.1.

### 4.3 Inflammatory and immune signaling

Pro-inflammatory signaling is a well-established trigger of Müller glia activation and arises from cytokines and growth factors released by neurons, vascular-associated cells, and other glia, particularly microglia (Kirsch et al., 2010; Wang et al., 2011). Müller glia are highly responsive to cytokines including TNFα and IL-6 family ligands, which activate pathways such as nuclear factor kappa B (NFκB) and Janus kinase/signal transducer and activator of transcription (JAK/STAT) signaling through gp130 (Kirsch et al., 2010). These inputs provide a direct route by which immune activation and tissue stress initiate Müller glia reactivity (Kirsch et al., 2010; Wang et al., 2011; Ji et al., 2022; Coorey et al., 2015).

Microglia–Müller glia crosstalk represents a central axis of this response. This interaction is bidirectional: microglia release signals, such as TNFα, that activate Müller glia, while activated Müller glia, in turn, release cytokines and other mediators that propagate inflammatory signaling across retinal layers and further influence microglia and the retinal vasculature (Wang et al., 2011; Ji et al., 2022). This coupling helps explain how localized immune activation can drive widespread Müller glia reactivity.

### 4.4 Müller glia–photoreceptor coupling

Müller glia are also tightly coupled to photoreceptors: their apical processes form adherens junctions with photoreceptor inner segments at the outer limiting membrane, and they provide the homeostatic and metabolic support that photoreceptor activity requires (Reichenbach and Bringmann, 2020; Hippert et al., 2015). As a result, Müller glia respond rapidly to photoreceptor stress by activating and remodeling (Leinonen et al., 2023). In light-induced photoreceptor degeneration, Müller glia express C-C motif chemokine ligand 2 (CCL2) within 12 h of exposure, most intensely around the developing lesion, before monocytes and microglia are recruited to the same region (Rutar et al., 2011). Intravitreal small interfering RNA (siRNA) knockdown of CCL2, which markedly reduced its expression in Müller glia, decreased immune cell infiltration of the outer nuclear layer and photoreceptor apoptosis (Rutar et al., 2012). Modulating Müller glia state has a similar effect: in Müller glia–photoreceptor co-cultures exposed to photo-oxidative damage, 670 nm photobiomodulation lowered Müller glia expression of CCL2, interleukin-1β (IL-1β), and IL-6, reduced the capacity of the culture medium to activate microglia and macrophages, and increased Müller glia mitochondrial membrane potential (Lu et al., 2017). Together, these studies support a feed-forward loop in which photoreceptor damage activates Müller glia, Müller glia–derived chemokines recruit immune cells, and the resulting inflammation accelerates photoreceptor loss. Because Müller glia responses to photoreceptor loss also differ between diseases, it will be important to define how this loop operates in AMD and inherited retinal degenerations.

### 4.5 Regulatory pathways controlling Müller glia reactivity

Beyond identifying activating inputs, it is critical to define the intracellular pathways that regulate Müller glia reactivity. Alongside NFκB, Activator Protein-1 (AP-1), and other stress-response pathways, one of the best-characterized signaling axes is the gp130–JAK–STAT3 pathway. Cytokines of the IL-6 family, including CNTF and LIF, signal through receptor complexes that recruit gp130, leading to activation of JAK kinases and phosphorylation of STAT3 (Kirsch et al., 2010). Phosphorylated STAT3 translocates to the nucleus and binds regulatory regions of target genes, including GFAP, promoting induction of intermediate filament proteins associated with gliosis (Coorey et al., 2015; Brenner and Messing, 2021) (Figure 3).

**FIGURE 3:**
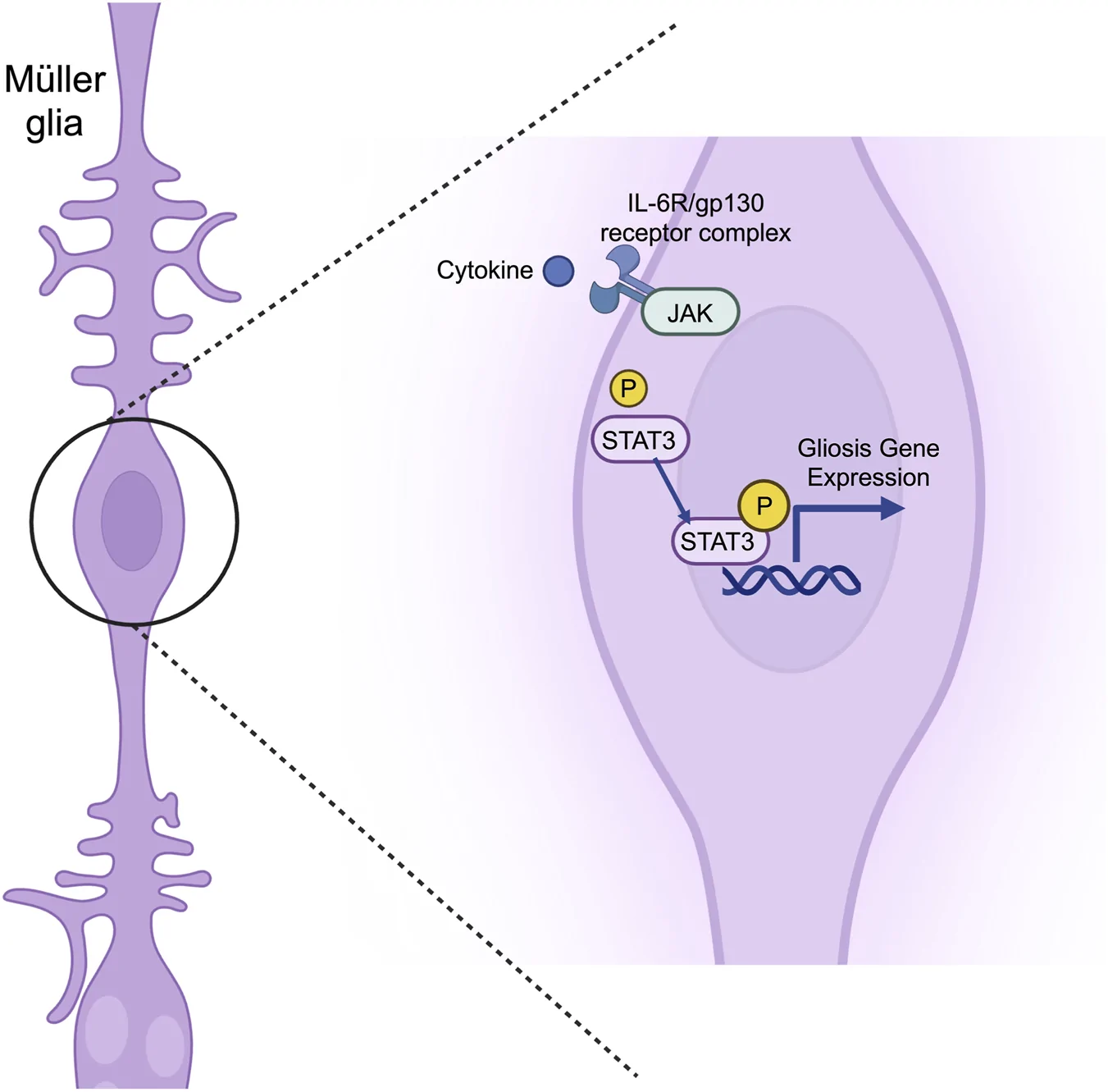
JAK/STAT3 signaling as a regulatory pathway controlling Müller glia reactivity. Schematic illustrating cytokine-driven activation of the JAK/STAT3 pathway in Müller glia. Binding of extracellular cytokines to the gp130/IL-6 receptor complex activates Janus kinase (JAK), leading to phosphorylation of signal transducer and activator of transcription 3 (STAT3). Phosphorylated STAT3 then translocates to the nucleus, where it promotes transcription of gliosis-associated genes, for example, GFAP (Graca et al., 2018; Brenner and Messing, 2021; Hagemann et al., 2023).

Importantly, Müller glia reactivity is not uniformly detrimental; early activation can be neuroprotective (Fischer and Bongini, 2010). Therapeutic strategies targeting this pathway will therefore likely need to modulate the magnitude and duration of signaling rather than eliminate it entirely. Together, these pathways suggest that Müller glia reactivity is best understood as a spectrum of state transitions that integrates diverse forms of retinal stress into shared but context-dependent outcomes.

## 5 Müller glia dysfunction in retinal disease

Prolonged Müller glia activation impairs core homeostatic functions, including neurotransmitter clearance, ion and water regulation, and neurovascular stability. Over time, sustained gliosis contributes to neuronal dysfunction and accelerates degeneration by increasing inflammatory mediator release and excitotoxic stress, and the magnitude and persistence of gliosis appear to increase with age (Graca et al., 2018). Below, we discuss how these changes manifest in glaucoma, diabetic retinopathy, and age-related macular degeneration (AMD).

### 5.1 Glaucoma: mechanical stress and RGC vulnerability

In glaucoma, progressive RGC loss is shaped by mechanical strain, metabolic stress, and neuroinflammation. While clinical therapies primarily lower IOP, glial reactivity and RGC degeneration often persist despite pressure reduction, highlighting the limits of targeting biomechanics alone (Fernández-Albarral et al., 2022; Inman and Horner, 2007). Müller glia are well positioned to sense IOP-related strain through mechanosensitive pathways and translate these cues into altered extracellular homeostasis and inflammatory signaling. In models such as the DBA/2J mouse, Müller glia gliosis (indicated by increased GFAP) occurs early, may persist after IOP normalizes, and scales with disease severity. This persistent gliosis is thought to compromise core homeostatic functions such as glutamate clearance and potassium buffering, which may elevate extracellular glutamate and potassium and increase excitotoxic stress on RGCs (Seitz et al., 2013; Jo AO. et al., 2022; Caldwell et al., 2023; Shinozaki and Koizumi, 2021). Whether Müller glia activation drives RGC injury or responds to early neuronal stress remains unresolved. The evidence favors bidirectional communication, and glaucoma progression can be framed as a positive feedback loop: stressed RGCs release injury signals that engage Müller glia, while sustained glial reactivity reshapes the synaptic environment to further compromise RGC survival.

### 5.2 Diabetic retinopathy and retinal ischemia

Although historically viewed as a vascular disease, diabetic retinopathy (DR) involves early and progressive neuronal dysfunction alongside vascular pathology, and Müller glia, which couple neurons and vasculature, are increasingly implicated in this progression (Carpi-Santos et al., 2022; Vujosevic and Midena, 2013; Fu et al., 2015). In diabetic models, reduced ERG b-wave amplitudes are consistent with impaired inner retinal signaling in Müller glia–regulated circuits (Fu et al., 2015). Diabetes and oxidative stress impair GLAST/EAAT1-dependent glutamate transport, increasing vulnerability to excitotoxic injury, while reduced Kir4.1 expression and altered aquaporin-4 function are associated with Müller glia swelling and retinal edema (Carpi-Santos et al., 2022; Yang et al., 2022; Coughlin et al., 2017). Müller glia also appear to actively contribute to vascular pathology. They are a major retinal source of VEGF under diabetic conditions, and conditional Müller glia VEGF knockouts show reduced leukostasis, tight junction loss, and vascular leakage relative to diabetic controls (Fu et al., 2015; Yang et al., 2022; Coughlin et al., 2017). This helps explain why anti-VEGF therapy reduces leakage while persistent glial dysfunction in neurotransmitter, ion, and metabolic support may continue to drive neurodegenerative features.

### 5.3 Age-related macular degeneration

AMD is characterized by progressive photoreceptor dysfunction and structural changes in the RPE and outer retina (Leinonen et al., 2023; Navneet et al., 2024; Zhao et al., 2024). Although Müller glia are less studied in AMD than in glaucoma or DR, their specialized macular organization, with distinct morphologies and higher densities supporting the metabolic demands of central vision, suggests they are important modulators of disease (Navneet et al., 2024; Zhao et al., 2024). Single-cell datasets implicate Müller glia in AMD-relevant pathways, including vascular signaling, extracellular matrix remodeling, and immune- and lipid-associated genes such as *APOE* and *CFI*, which also links Müller glia to the lipid handling discussed in Section 6.1 (Leinonen et al., 2023; Navneet et al., 2024; Zhao et al., 2024; Zeng et al., 2025). In injury-based AMD models, subpopulations with distinct stress-response programs emerge, suggesting that altered metabolic support and inflammatory signaling within the macular niche may contribute to disease progression.

### 5.4 Shared features of Müller glia dysfunction across diseases

Across these disease contexts, Müller glia dysfunction shows both shared and disease-specific features. Early responses may be adaptive, helping preserve retinal structure and homeostasis through trophic, anti-inflammatory, metabolic, and structural support. With chronic stress, however, these responses can shift toward sustained gliosis and progressive disruption of core homeostatic functions.

At the same time, Müller glia reactivity is not expressed uniformly across retinal disease. Similar markers of gliosis are observed across disorders, yet dominant pathological outcomes differ, including RGC loss in glaucoma, vascular dysfunction in diabetic retinopathy, and photoreceptor degeneration in AMD and inherited retinal disease (Table 2). This divergence suggests that Müller glia responses are shaped by the nature and location of the primary insult and may be spatially compartmentalized across retinal layers. Defining how these disease-specific Müller glia states emerge will be important for determining when Müller glia preserve retinal resilience and when they contribute to degeneration (Hippert et al., 2015).

**TABLE 2:** Shared and disease-weighted features of Müller glia dysfunction in glaucoma, diabetic retinopathy, and AMD.

​	Glaucoma	Diabetic retinopathy	AMD
Reactive gliosis	Prominent	Prominent	Prominent
Glutamate dysregulation	Strong	Moderate	Context dependent
Ion and water imbalance	Moderate	Strong	Moderate
Metabolic and redox stress	Strong	Strong	Strong
Vascular barrier dysfunction	Moderate	Strong	Moderate to strong, depending on subtype
Inflammation	Strong	Strong	Strong

This table provides a comparative summary of Müller glia dysfunction across three common retinal diseases. Across glaucoma, diabetic retinopathy, and age-related macular degeneration (AMD), Müller glia exhibit overlapping patterns of reactive gliosis, inflammatory activation, metabolic and redox stress, and disruption of neurotransmitter, ion, water, and vascular homeostasis. At the same time, the relative prominence of these features varies by disease context, reflecting differences in initiating stressors, vascular involvement, and tissue compartment affected (Seitz et al., 2013; Leinonen et al., 2023; Inman and Horner, 2007; Vujosevic and Midena, 2013; Fu et al., 2015; Navneet et al., 2024; Zeng et al., 2025).

## 6 Therapeutic modulation of Müller glia reactivity states

Because chronic Müller glia dysfunction contributes to neuronal, metabolic, and vascular pathology across retinal disease, strategies that selectively modulate Müller glia state have emerged as a promising therapeutic direction. Rather than simply suppressing gliosis, emerging strategies aim to shift Müller glia toward more homeostatic states while limiting maladaptive inflammatory and metabolic responses. These approaches include pharmacological modulation of glial signaling pathways (including lipid metabolism and signaling), restoration of ion and metabolic homeostasis, and gene-based or regenerative interventions targeting Müller glia directly.

### 6.1 Protective lipid signaling and anti-inflammatory reprogramming

While bioactive lipid mediators can actively resolve existing inflammation, the baseline metabolic and structural lipid state of Müller glia may also influence their threshold for reactivity. Rather than viewing lipid alterations solely as downstream consequences of injury, emerging evidence suggests that lipid and metabolic homeostasis could prime Müller glia toward resilience or maladaptive gliosis, although direct tests of this causal direction remain limited. Lipid biology can influence Müller glia at three levels: sterol handling, membrane composition, and bioactive mediator signaling.

Müller glia are thought to be major contributors to cholesterol homeostasis in the neural retina, as astrocytes are in the brain, supporting neuronal sterol supply through ABCA1-dependent efflux of ApoE-containing lipoproteins (Ramachandra Rao and Fliesler, 2021). Excess cholesterol is eliminated in part as oxysterols, and Müller cells also respond to neuron-derived oxysterols such as 24S-hydroxycholesterol, which stimulates ABCA1 and ApoE expression in Müller cell cultures (Masson et al., 2024). Oxysterols can act as LXR ligands that promote cholesterol efflux and repress NFκB-driven inflammatory transcription. They are not uniformly protective, however, since some oxysterols, such as 7-ketocholesterol, are cytotoxic and pro-inflammatory.

Membrane lipid composition may add a further layer. Cholesterol- and sphingolipid-enriched microdomains organize surface receptors and ion channels in many cell types, and changes in lipid saturation and cholesterol content alter membrane fluidity and domain organization (Campbell et al., 2022). Because Müller glia rely on mechanosensitive channels such as TRPV4 and on cytokine receptors, it is plausible that altered membrane lipid composition shifts the threshold for mechanical or inflammatory activation. This has not been directly tested in Müller glia.

Building on this metabolic landscape, specialized pro-resolving mediators (SPMs), including lipoxins, resolvins, protectins, and maresins, are endogenous bioactive lipids generated from polyunsaturated fatty acids that have anti-inflammatory actions, help terminate inflammatory responses, and promote tissue repair (Wei and Gronert, 2019; Wei and Gronert, 2017). In the retina, lipoxin pathways have been linked to neuroprotection and regulation of glial inflammatory states: lipoxins protect RGCs in injury and glaucoma-related contexts, and multiple studies have shown direct effects of lipoxins on retinal glial activation (Wei and Gronert, 2019; Wei and Gronert, 2017).

Amplifying lipid signaling in Müller glia can reshape inflammatory tone in more than one direction, making these pathways attractive but complex leverage points. For example, cytochrome P450 epoxygenase-derived epoxidated fatty acids suppress Müller glia inflammatory responses, while RvD1 reduces inflammatory and VEGF-associated stress responses under high-glucose conditions but has also been associated with Müller glia hypertrophy in some settings (Chen et al., 2022). These observations suggest that anti-inflammatory lipid signaling does not simply suppress gliosis but modulates the qualitative state of glial responses.

Within this landscape, the LXB_4_ pathway has emerged as a candidate link between neuroprotective signaling and regulation of Müller glia activation state. Early work identified LXB_4_ as part of an astrocyte-derived neuroprotective signal in the inner retina, and more recent studies identify Müller glia as a key cellular source of LXB_4_ in the retina (Kumar et al., 2026; Livne-Bar et al., 2017; Karnam et al., 2024; Maurya et al., 2024; Figure 4). In mouse ocular hypertension (OHT), amplifying retinal LXB_4_ signaling was associated with lower gliosis markers such as GFAP and reduced inflammatory outputs, including during TRPV4-driven activation (Karnam et al., 2024).

**FIGURE 4:**
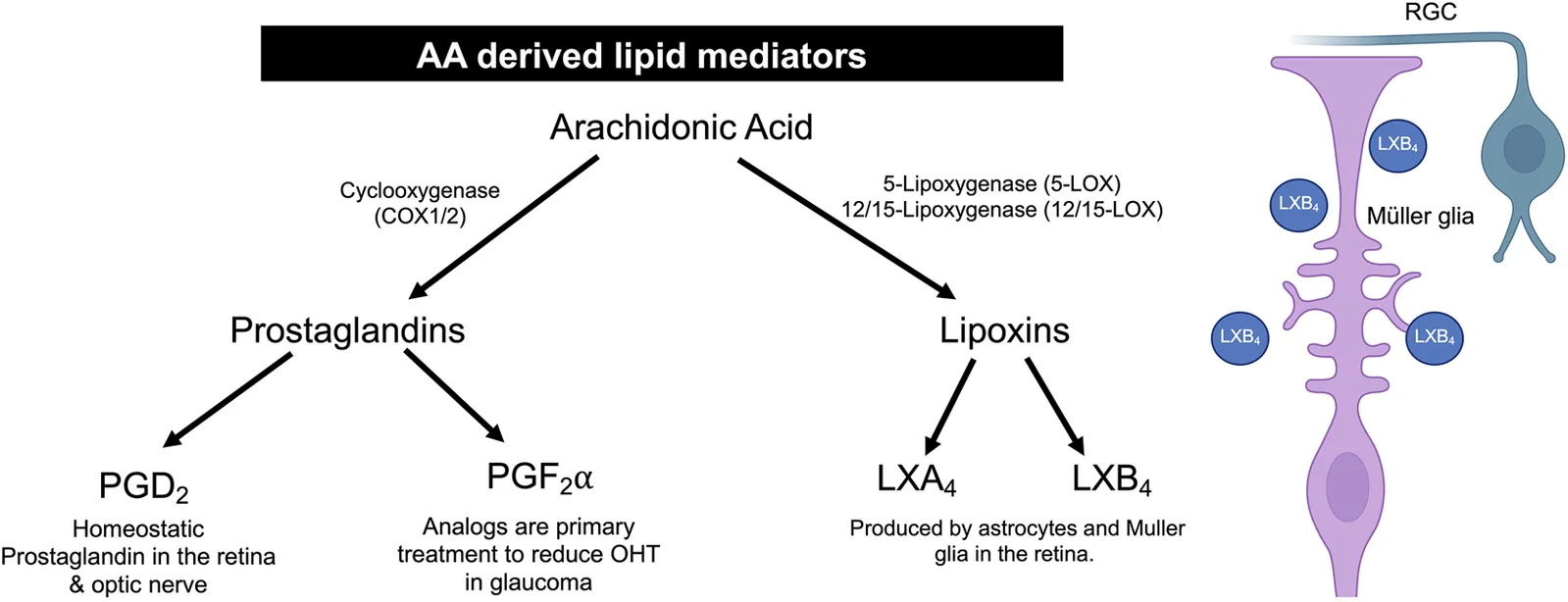
Arachidonic acid-derived lipid mediator pathways with emphasis on lipoxin biosynthesis in Müller glia. Schematic overview of arachidonic acid metabolism. The left panel summarizes the broader arachidonic acid cascade, in which arachidonic acid can be metabolized through cyclooxygenase (COX-1/2) pathways to generate prostaglandins or through lipoxygenase (LOX) pathways, including 5-LOX and 12/15-LOX, to generate lipoxins. Schematic of Müller glia-derived LXB_4_ signaling to retinal ganglion cells (RGCs) (Wei and Gronert, 2019; Wei and Gronert, 2017; Kumar et al., 2026; Karnam et al., 2024; Maurya et al., 2024; Ward et al., 2026; Livne-Bar et al., 2024).

The translational path for LXB_4_-based therapeutics remains at an early stage: receptor mechanisms are not fully defined, most evidence comes from preclinical models, and ocular delivery and analog design are still in development. These findings nonetheless support a therapeutic approach aimed at shifting macroglial inflammatory reactivity rather than broadly suppressing it. Anti-VEGF therapies and corticosteroids reduce vascular leakage and inflammation but do not directly target the upstream state of glial reactivity (Chen et al., 2022). Endogenous lipid modulators such as LXB4, potentially combined with strategies that support membrane composition and cholesterol efflux, could offer a way to promote return to homeostasis while avoiding broad immunosuppression. The latter combination is, at present, a hypothesis.

### 6.2 Restoring ion channel and metabolic homeostasis

Restoring Müller glia ion-channel and calcium homeostasis has emerged as a promising strategy to reduce chronic gliosis and protect retinal neurons. TRPV4 is a mechanosensitive and osmosensitive channel expressed in Müller glia that couples tissue stress to calcium influx and inflammatory signaling (Jo AO. et al., 2022; Jo et al., 2015). In retinal explants, pharmacologic inhibition of TRPV4 improves RGC survival and attenuates degenerative changes (Taylor et al., 2017). In Müller glia, TRPV4 activation engages gliosis-associated programs and inflammatory outputs, and statins suppress TRPV4-associated inflammatory signaling, including NFκB and TNFα-linked pathways, suggesting an indirect route to reduce glial inflammatory amplification (Jo AO. et al., 2022; Jo YH. et al., 2022). Endogenous pathways may also converge on this channel: in a mouse model of glaucomatous injury, OHT triggers a pronounced increase in Müller glia TRPV4, and LXB_4_ treatment inhibits this upregulation at both the RNA and protein level (Jo YH. et al., 2022). This suggests that amplifying endogenous protective pathways such as LXB_4_ could help restore a homeostatic Müller glia phenotype.

TRPV4 signaling intersects with volume regulation pathways that contribute to edema and barrier stress (Chen et al., 2024). Edema-related injury can activate TRPV4-dependent calcium signaling and inflammatory mediator release (Jo et al., 2015). In DR models, AQP4 and TRPV4 expression increase under high-glucose conditions, and AQP4 inhibition reduces ferroptosis and oxidative stress *in vitro* while alleviating retinal injury *in vivo*, in part through downregulation of TRPV4 (Kumar et al., 2026). However, these data remain largely preclinical and highly model-dependent, and the relative contribution of TRPV4/AQP4 signaling may differ across diseases, stages, and injury contexts. As a result, the translational relevance of these pathways will require clearer validation in disease-specific settings.

### 6.3 Gene therapy and cell-specific targeting strategies

Müller glia are attractive targets for retinal gene therapy because they contact major neuronal classes, interface with retinal vasculature, and can influence broad retinal regions from a relatively small population of cells. In principle, this anatomy makes them well suited for delivery strategies aimed at restoring retinal homeostasis or providing sustained local expression of therapeutic factors.

Progress in Müller glia targeting has been driven by capsid engineering and promoter selection in preclinical systems. Directed evolution identified the ShH10 capsid, an AAV6-related variant capable of efficient and selective intravitreal transduction of rat Müller glia, with improved performance relative to commonly used serotypes. Subsequent work identified ShH10Y, AAV9, and regulatory elements such as the RLBP1 promoter as useful tools for Müller glia-biased expression in experimental models (Klimcz et al., 2009). Disease-directed *in vivo* studies further support the relevance of this approach. In CRB1 retinitis pigmentosa animal models, co-targeting Müller glia and photoreceptors improved retinal structure and function, whereas restricting expression to a single cell type was insufficient. Macroglia-directed AAV strategies have also been used to deliver therapeutic enzymes that amplify lipoxin formation, including a macroglia-targeted AAV-human 15-LOX construct that protects against ocular hypertension–associated neurodegeneration and preserves ganglion cell complex integrity (Maurya et al., 2025).

At the same time, important translational limitations remain. Capsid tropism and promoter performance differ across species, and approaches that work efficiently in rodents may not generalize directly to primate or human retina. Delivery route also matters; intravitreal access is attractive but may not uniformly reach Müller glia across disease states, whereas subretinal delivery introduces different anatomical and procedural constraints. In addition, Müller glia accessibility and transduction efficiency may change with disease stage as retinal architecture, barrier properties, and gliotic state evolve. Promoter specificity is also relative rather than absolute, and markers such as RLBP1 may not provide identical selectivity across species and conditions. Together, these issues suggest that Müller glia–targeted gene therapy is a promising preclinical strategy, but one whose translational performance will depend heavily on disease context, vector design, and delivery paradigm.

### 6.4 Müller glia reprogramming and regenerative potential

Müller glia reprogramming remains an appealing therapeutic concept because these cells arise from retinal progenitor lineages and retain latent developmental programs that can be re-engaged under some conditions (Ahmad et al., 2011; Goldman, 2014; Gao et al., 2021). This potential is most clearly demonstrated in zebrafish, where Müller glia respond to injury by re-entering the cell cycle and generating progenitor-like cells capable of differentiating into multiple retinal neuron types. In mammals, however, endogenous reprogramming remains limited, particularly in the adult retina. Although factors such as Achaete-scute family bHLH transcription factor 1 (ASCL1), Lin-28 homolog (LIN28), and Heparin binding EGF like growth factor (HB-EGF) can partially unlock neurogenic potential under permissive conditions, these interventions have not yet achieved robust or reliable regeneration in mature mammalian tissue (Goldman, 2014; Gao et al., 2021).

The major barriers are not simply initiation of reprogramming, but functional translation. Reprogrammed cells must generate the appropriate neuronal subtypes, integrate into existing retinal circuits, survive within an inflammatory or degenerative environment, and do so with sufficient efficiency and safety to be therapeutically meaningful (Salman et al., 2021; Guo et al., 2023). These limitations currently make Müller glia reprogramming better suited as a mechanistic framework for regenerative biology than as a near-term translational strategy. Continued comparative work will be essential for identifying which features of regenerative competence can be realistically transferred to the adult mammalian retina (Fischer and Reh, 2003; García-García et al., 2020).

## 7 Discussion: experimental approaches, current limitations, and future directions

Müller glia state is best viewed as the product of several layers (transcriptional, epigenetic, post-transcriptional, metabolic, and spatial), each captured by a different technology. Recent advances in Müller glia enrichment and single-cell sequencing now resolve Müller glia responses into distinct, multi-layered cell states across species and disease models. Rather than a single uniform gliotic state, single-cell RNA sequencing (scRNA-seq) reveals that mammalian Müller glia adopt highly heterogeneous, context-dependent reactive profiles in response to injury (Yao et al., 2024). In regenerative systems such as zebrafish, these approaches identify clear state transitions that span resting, activated, and proliferative programs. By mapping this foundational transcriptional layer, these datasets help associate specific stressors with distinct Müller glia gene programs, distinguishing cell-intrinsic responses from secondary changes shaped by surrounding immune and vascular environments.

Multi-omic approaches extend these insights by defining the regulatory layers operating beyond transcript abundance. Chromatin-accessibility profiling, including scATAC-seq, defines the epigenetic layer, demonstrating how Müller glia competence and injury responsiveness are either constrained or enabled by their chromatin state (Norrie et al., 2025; Grosche et al., 2016). Cross-species comparisons further suggest that regenerative divergence may reflect differences in transcription factor networks and enhancer usage rather than fundamental differences in cellular architecture. In parallel, proteomic and lipidomic strategies capture the post-transcriptional and bioactive signaling layers, including sterol, membrane, and mediator lipids such as LXB_4_, which cannot be inferred from RNA alone and which the lipid-state hypothesis in Section 6.1 predicts may set reactivity thresholds.

Crucially, the emerging application of spatial omics approaches provides a novel layer of anatomical context, mapping these distinct Müller glia states directly within the intact retinal architecture. Because Müller glia span all retinal laminae and their functions are highly compartmentalized, spatial transcriptomics and multiplexed spatial proteomics allow researchers to link specific molecular states to localized retinal microenvironments. This spatial resolution is vital for determining whether particular reactive profiles, such as specific immune-regulatory or vascular-supporting states, arise uniquely at the inner blood-retinal barrier or adjacent to injured photoreceptors in the outer retina, bridging the gap between single-cell sequencing and functional tissue pathology (Storey et al., 2026; Schumann et al., 2025).

Human retinal organoid systems provide experimentally tractable platforms for testing Müller glia-derived therapeutic signals. For example, in human embryonic stem cell-derived retinal organoids, RGC-like cells exposed to NMDA show reduced neurite length that can be rescued by treatment with Müller glia-derived extracellular vesicles (Eastlake et al., 2026). Together, these approaches are refining how Müller glia states are defined and compared across experimental systems.

### 7.1 Experimental limitations in defining Müller glia function

Despite these advances, important technical limitations still complicate interpretation of Müller glia function. Although Müller glia are morphologically distinctive in intact retina, assigning specific functions to Müller glia versus other glia remains challenging. Müller glia and retinal astrocytes share overlapping roles in neurotransmitter clearance, ion and water homeostasis, and neurovascular support. As a result, changes in glutamate handling, potassium buffering, or vascular stability in disease models cannot always be attributed to a single glia type without cell-type–resolved tools and readouts.

Technical limitations further complicate interpretation. Retinal dissociation disrupts Müller glia processes and can induce stress responses, and enrichment strategies such as FACS often trade cellular purity for altered cellular state. Primary Müller glia cultures can reach high enrichment but commonly retain low-level contamination from other glia, including astrocytes, and culture conditions can shift glia phenotypes. Marker ambiguity is an additional barrier. Many widely used Müller glia markers, including glutamine synthetase (GS), SOX9, vimentin, and nestin, are not strictly Müller-specific and can overlap with astrocytes or reactive glia states (Peng et al., 2013). Conversely, classic astrocyte markers such as GFAP and S100B are not exclusive astrocyte markers under injury conditions because reactive Müller glia also induce these proteins. RLBP1 is often used to enrich Müller glia identity but can show expression in other retinal cell types depending on species and context, underscoring the need for multi-marker definitions rather than single-marker assignment (Table 3).

**TABLE 3:** Comprehensive List of Müller Glia Markers.

Gene/Marker	Localization	Specificity	Homeostatic/Reactivity marker
Glutamine synthetase (GS)	Cytosol	Strong MG marker, astrocytes	Homeostatic marker
CRALBP/RLBP1	Cytoplasm	Strong MG marker, RPE cells	Homeostatic marker (potentially reactive)
GLAST/EAAT1	Cell surface	Strong MG marker, astrocytes	Homeostatic marker
Vimentin	Intermediate filament/cytoskeleton	Strong MG marker, astrocytes, endothelial cells	Reactivity marker
Aquaporin-4	Plasma membrane	MG marker, astrocytic/glia cells in CNS	Homeostatic marker
Kir4.1	Plasma membrane	MG marker, astrocytic/glia cells in CNS	Homeostatic marker
Clusterin/Clu	Cytoplasm	MG marker, astrocytic/glia cells in CNS	Reactivity marker
Apolipoprotein E	Cytoplasm	MG marker, astrocytes, microglia/macrophages, RPE	Reactivity marker
SOX9	Nucleus	MG marker, CNS glia lineages	Reactivity marker
CD44	Cell surface	Strong MG marker, broadly expressed by immune cells	Reactivity marker
GFAP	Intermediate filament cytoskeleton	Strong MG marker, astrocytes	Reactivity marker
Nestin	Intermediate filament cytoskeleton	Strong MG marker, astrocytes, endothelial/mesenchymal cells	Reactivity marker
ALDH1L1	Cytosol	Strong MG marker, astrocyte marker in CNS	Homeostatic marker
S100β	Cytosol/nucleus	MG marker, astrocytes, RPE	Reactivity marker

Summary of commonly used molecular markers for identifying Müller glia in the retina. Markers are listed with their primary cellular localization and their relative specificity for Müller glia. Markers are further categorized as homeostatic or reactive based on their predominant use in the literature (Ning et al., 2022; Bringmann et al., 2006; Roesch et al., 2008).

These limitations underscore the importance of approaches that preserve spatial and cellular context. Single-cell and spatial transcriptomics, lineage-based genetic strategies, and *in vivo* imaging provide complementary ways to distinguish intrinsic Müller glia programs from secondary changes driven by immune or vascular remodeling. Linking these approaches to functional assays will be essential for determining what specific Müller glia phenotypes and transitions drive retinal disease or restore homeostasis.

### 7.2 Future directions: toward functional and translational readouts

The field now needs to determine how specific Müller glia states affect retinal physiology *in vivo*. For Müller glia, the most informative studies will be those that connect molecular state changes to retinal physiology using measures such as ERG, OCT-based structural analysis, vascular permeability assays, glia swelling and osmotic stress readouts, and behavioral measures of visual function, ideally within the same model and across matched disease stages.

Equally important is the need to localize Müller glia states and phenotypes within intact tissue. Because Müller glia are anatomically compartmentalized, this spatial context will be critical for understanding how a given molecular program translates into circuit instability, barrier dysfunction, or impaired metabolic support. Retinal organoids and *ex vivo* systems can complement these approaches by allowing controlled perturbation of specific inputs such as hypoxia, mechanical strain, or inflammatory cytokines.

Taken together, the most useful future framework is not simply to identify more Müller glia states, but to determine which states arise where, what they do, and whether they can be selectively shifted without disrupting essential homeostatic support. Resolving those questions will be critical for translating Müller glia state regulation into tractable therapeutic strategies.

## 8 Conclusion

Müller glia are central regulators of retinal homeostasis, integrating structural, metabolic, synaptic, and vascular support across the full depth of the retina. Their responses to stress are increasingly recognized not as secondary features of disease, but as dynamic state transitions that can initially preserve tissue function and, when sustained, drive chronic neuroinflammation, neurovascular dysfunction, and neuronal degeneration. Across glaucoma, diabetic retinopathy, AMD, and other retinal disorders, Müller glia emerge not as a uniform reactive population, but as context-dependent responders whose outputs are shaped by the nature, location, and duration of injury.

This perspective reframes Müller glia reactivity as a process to be precisely modulated rather than broadly suppressed. A central challenge for the field is to define which Müller glia programs are protective, which are maladaptive, and when these transitions occur during disease progression. Advances in single-cell and spatial omics, together with cell-specific perturbation strategies and *in vivo* functional readouts, now provide the tools to resolve these questions with increasing precision.

Looking forward, integrating molecular state definitions with functional and spatial context will be essential for translating Müller glia biology into targeted therapies. Defining how distinct Müller glia states map onto specific retinal circuits, vascular niches, and disease stages will help identify tractable intervention points. In this framework, Müller glia are not only indicators of retinal stress, but also important regulators of disease progression and promising therapeutic entry points for restoring retinal homeostasis, limiting neurodegeneration, and promoting resilience in the diseased retina.

## Glossary

ABCA1: ATP-binding cassette transporter A1
AMD: age-related macular degeneration
ApoE: apolipoprotein E
AQP4: aquaporin-4
BRB: blood–retinal barrier
CCL2: C-C motif chemokine ligand 2
CNS: central nervous system
CNTF: ciliary neurotrophic factor
DR: diabetic retinopathy
EAAT1: excitatory amino acid transporter 1
ERG: electroretinogram/electroretinography
FGF2: fibroblast growth factor 2
GFAP: glial fibrillary acidic protein
GLAST: glutamate/aspartate transporter
GS: glutamine synthetase
IL-1β: interleukin-1 beta
IL-6: interleukin-6
IOP: intraocular pressure
JAK: Janus kinase
Kir4.1: inwardly rectifying potassium channel 4.1
LIF: leukemia inhibitory factor
**LXB**_**4**_: Lipoxin B_4_
LXR: liver X receptor
NFκB: nuclear factor kappa B
OCT: optical coherence tomography
OHT: ocular hypertension
RGC: retinal ganglion cell
RLBP1: retinaldehyde-binding protein 1
ROS: reactive oxygen species
RPE: retinal pigment epithelium
siRNA: small interfering RNA
scRNA-seq: single-cell RNA sequencing
SPMs: specialized pro-resolving mediators
STAT3: signal transducer and activator of transcription 3
TNFα: tumor necrosis factor alpha
TRPV4: transient receptor potential vanilloid 4
VEGF: vascular endothelial growth factor
